# Study Abroad Angst: A Literature Review on the Mental Health of International Students During COVID-19

**DOI:** 10.3390/ijerph21121562

**Published:** 2024-11-26

**Authors:** Daisuke Akiba, Michael Perrone, Caterina Almendral

**Affiliations:** 1 School of Education, Queens College & The Graduate Center, The City University of New York, Queens, New York, NY 11367, USA; 2 School of Education, Queens College, The City University of New York, Queens, New York, NY 11367, USA; michael.perrone@qc.cuny.edu; 3 LaGuardia Community College, The City University of New York, Long Island City, NY 11101, USA; calmendral@lagcc.cuny.edu

**Keywords:** COVID-19, depression, higher education, international students, mental health, pandemic, psychological distress, student mental health, thematic analysis

## Abstract

Background: The COVID-19 pandemic presented unique and unprecedented challenges for international students, those studying at institutions of higher education outside of their home countries, due to their distinct circumstances and vulnerabilities. This literature review examines the multifaceted mental health burdens they experienced and highlights the need for targeted support and interventions. Methods: A rigorous search across three databases (i.e., PubMed, PsycINFO, and ERIC) yielded 50 empirical studies for inclusion in this literature review. A six-phase thematic analysis framework was employed to identify and synthesize key themes. Results: Seven prominent themes emerged: (1) academic and professional disruptions; (2) challenges navigating international student status; (3) social isolation and loneliness; (4) difficulties with living arrangements; (5) financial and food insecurity; (6) health concerns for self and loved ones; and (7) experiences of discrimination and xenophobia. Conclusions: This review highlights a range of tolls that mental health consequences took on international students, and it suggests the need for targeted interventions and support services to address these challenges. It also identifies critical research gaps, such as the need for longitudinal studies and comparative analyses with domestic students. The implications for inclusive policies and supportive environments to promote international students’ well-being are discussed.

## 1. Introduction

As the world grappled with the COVID-19 pandemic, an unprecedented crisis in modern history, a particular segment of the academic community found themselves at the epicenter of a unique set of challenges. International students—individuals studying at institutions of higher education located in countries other than their own—faced multifaceted hardships during and following the pandemic [1,2]. Specifically, amidst the global health crisis, these students navigated not only the typical adversities associated with pursuing education in a foreign environment, such as linguistic barriers and cultural adjustments [3,4], but also the heightened uncertainties surrounding the global pandemic, the increased risk of infections, and unfamiliar arrangements such as travel restrictions and social distancing [5].

The World Health Organization officially classified COVID-19 as a global health pandemic on 11 March 2020 [6]. In response, there was a universal trend of abrupt suspension of in-person activities and services at institutions of higher education and eventual conversion to fully online instructional and service delivery, impacting all learners—domestic and international [7,8,9,10]. Additionally, numerous countries immediately declared states of national health emergency and swiftly imposed widespread lockdowns [11]. Governments around the globe enforced strict measures such as international travel bans and changes to student-visa-related rules [12]. Consequently, international students faced additional layers of stressors compared to their domestic counterparts, potentially translating into heightened vulnerability to pandemic-related mental health issues [13,14]. It is therefore reasonable to presume that the COVID-19 pandemic’s impact was likely more profound on international students than on domestic university students [15]. Thus, it appears imperative that we examine the ramifications of the COVID-19 pandemic on the mental health of this specific population.

While there is a growing body of research examining the impact of the COVID-19 pandemic on the broader university student population [16,17], the focus on international students as a specific unit of analysis has been relatively limited. Furthermore, the target populations, research methodologies, settings, and other variables exhibit significant diversity among studies that do focus on international students, rendering a systematic understanding of the topic challenging. It may thus be unsurprising that while some researchers report international students to have suffered a sharper decline in mental health compared to their domestic counterparts during the pandemic [18,19,20], others have reported the contrary [21,22], as described in more detail in later sections. The current review of the literature, therefore, has been crafted with the objective of illuminating the profound, contextualized, and multifaceted repercussions of the COVID-19 pandemic for international students, thereby promoting a more systematic understanding of its mental health impact. Specifically, it is essential to examine the potential for heightened levels of anxiety, depression, and stress among international students due to the unique challenges they faced during the pandemic. Understanding the implications of these experiences for students’ mental health and academic success is crucial to developing effective support measures and interventions.

In alignment with the conceptualizations prevalent in the literature reviewed, we define psychological well-being, in the context of this review, as a state of mental health characterized by the absence of unusual depression, anxiety, or distress, beyond what individuals may ordinarily experience. Psychological well-being, furthermore, encompasses a state of mental health characterized by minimal and manageable distress, as well as resilience, emotional stability, and social connectedness [23,24]. During the COVID-19 pandemic, these facets were often compromised due to external stressors unique to the international student experience, including social isolation, academic disruptions, and uncertainty regarding visa and residency status, as described in more detail in this review [25,26]. There are several core reasons pointing to the critical importance of exploring the psychological well-being of international students studying at institutions of higher education away from their home countries—particularly as the world continues to recover from the unprecedented collective trauma of the COVID-19 pandemic. Through a comprehensive review of existing research on this topic in the post-pandemic world, we can shed light on the mental health challenges faced by stranded international students across different regions—which, in turn, will inform us of the specific need to provide interventions as appropriate for the impacted students as well as future international students, should crises of a similar nature transpire. This is particularly pertinent, given that data have emerged indicating that the adverse psychological impact of the pandemic may endure on a long-term basis [27].

At a more societal level, university students studying abroad have been shown to enrich the experiences, perspectives, knowledge, and skills, not only among the international students themselves but also among the students, faculty, and other members of the host institutions at large [28]. In the same vein, international students can serve the local communities effectively, as well; specifically, Akiba [29] reports a successful social justice-driven English as a second language program in which international students worked with children at a neighboring homeless shelter, providing academic and social mentorship transcending the linguistic and other cultural barriers. Finally, in today’s increasingly global society, encouraging university students to study abroad represents an effective way of building future international leaders, while fostering a climate grounded in intercultural collaboration [30].

Thus, it is in the best interest not only of individual international students but also of entire society for us to examine the nature of their experiences and ensure the well-being of these students. The results of this investigation hold the potential to inform targeted public health interventions, tailored to address the specific mental health needs of international students facing protracted isolation during the COVID-19 pandemic.

As discussed in more detail in later sections, the current review reveals that the COVID-19 pandemic presented international students with a unique constellation of mental health challenges across multiple domains. The key themes include academic disruptions, uncertainties surrounding visa and residency status, heightened social isolation, difficulties with living arrangements, financial and food insecurity, concerns about personal and familial health, and experiences of discrimination and xenophobia. These interconnected challenges underscore the vulnerability of international students during the pandemic, with each factor contributing to an increased risk of psychological distress. This review employs a thematic analysis to provide an in-depth exploration of these challenges and their impact on mental health.

## 2. Methods

### 2.1. Overview

The goal of this review is to shed light on the complex and multifaceted mental health consequences of the COVID-19 pandemic for international students, contributing to a more synthesized and systematic understanding of this issue. To this end, a comprehensive literature search was conducted in three major databases, as described in more detail below, in June 2024. Upon completion of the initial search process, duplicates were removed, the abstracts were reviewed for relevance and appropriateness as detailed and, in the end, 50 articles were included in the analysis as follows.

### 2.2. Search Strategy

To comprehensively explore the literature on the psychological well-being of international students during the COVID-19 pandemic, we conducted a targeted search across three prominent databases: PubMed, PsycINFO, and ERIC. These databases were strategically selected due to their explicit relevance to the research question and are recognized as leading resources in their respective fields, so they would add up to a comprehensive and cohesive whole in the context of public health’s interdisciplinary nature. PubMed provides extensive coverage of the biomedical and health literature, capturing studies on the physical and mental health impacts of the pandemic. PsycINFO offers a comprehensive repository of psychological research, including studies on stress, coping, and well-being. ERIC focuses specifically on the education-related literature, including issues pertaining to the world of higher education. This focused approach ensured the retrieval of studies directly addressing the intersection of international students’ mental health, educational experiences, and the COVID-19 pandemic. The inclusion of broader databases like ProQuest and EBSCO was initially considered; however, they did not add anything noticeable to the searches based on the three specialized databases and thus were deemed unnecessary for this review. The search was commenced in March 2024 and the ongoing update process lasted through June 2024, capturing studies published beginning in March 2020, when COVID-19 began to affect schools across the globe [31].

The search strategy incorporated specific keywords and phrases to retrieve relevant studies, ensuring a thorough examination of the topic. The “wildcard” feature was utilized to encompass variations in word endings and to account for singular/plural forms. For example, “international studen*” and “foreign studen*” were used to capture the variability in word endings associated with “student”. Similarly, “depress*” was used with an intent to capture the literature with keywords such as “depression”, “depressed”, “depressive”, and other possible derivatives. The search strategy combined three dimensions of keywords with the logical operator “AND” to ensure the retrieval of relevant articles. This resulted in 40 unique combinations (2 terms × 4 terms × 5 terms) of the search terms, as follows:

- Two terms for the population:
  - international studen*
  - foreign studen*
- Four terms for the circumstance:
  - pandemic
  - COVI*
  - Coronavirus
  - SARS-CoV-2
- Five terms concerning the mental-health-related dimensions:
  - depress*
  - stres*
  - anxiety
  - psychological
  - mental health

Given that the pandemic was not declared until March 2020, the publication date range was set to begin in 2020, with no specified end date. This rigorous database search approach aimed to gather the pertinent literature on the mental health status of international students during the COVID-19 pandemic, facilitating a comprehensive analysis of the subject matter.

In addition to the systematic searches conducted in electronic databases, a supplementary manual search of relevant journals, articles, and references cited within the initially retrieved articles was performed. This approach aimed to ensure a comprehensive exploration of the literature related to the subject matter on the topic. By combining both database-driven and manual search methods, the study’s search strategy was designed to maximize the inclusiveness and depth of the information gathered, contributing to the robustness of the findings presented in this peer-reviewed publication. This process yielded a final pool of 266 articles, including duplicates, for further screening.

### 2.3. Inclusion and Exclusion Criteria

Although this comprehensive literature review was not intended to be a systematic review or meta-analysis, we made a concerted effort to follow rigorous screening standards in the selection of the relevant literature to ensure that the pool of literature analyzed would be optimal. All 266 articles screened through the initial search results underwent a thorough assessment for relevance, primarily based on their abstracts, aligning with the key objectives of this comprehensive literature review. The following inclusion criteria were developed to ensure that the selected studies provided empirical results that pertain to the mental health impact of the pandemic on international students. Specifically, for inclusion, we sought the following:

- Studies that directly examined mental health outcomes during the COVID-19 pandemic, such as depression, anxiety, and psychological stress, empirically among international higher education students.
- Empirical research that treated international students as a distinct group.
- Peer-reviewed articles published in English between March 2020 and June 2024.

The exclusion criteria, by contrast, were as follows:

- Research that did not articulate the COVID-19 pandemic as a focus when discussing mental health.
- Studies that did not provide sufficient differentiation between international and domestic university students (e.g., studies that noted that the participants included both domestic and international students but did not differentiate them otherwise when reporting results).
- Non-empirical publications (e.g., perspective papers, editorials, letters to the editor, etc.).
- Studies that did not explicitly analyze dimensions concerning mental health among the variables.

By employing this exclusion process, the current research ensured the incorporation of studies that comprehensively addressed mental health issues in the context of the COVID-19 pandemic, specifically targeting international students as the subject of interest.

### 2.4. Results of the Literature Screening

The initial database search using the 40 predefined search terms retrieved 257 citations across the three databases. Some articles appeared multiple times, both within and across databases, due to overlapping indexing. In addition, 9 articles that were frequently cited during the screening process but did not appear in the database search results were manually added, bringing the total to 266 potential articles. After the removal of duplicates, 93 unique articles remained for further screening. A detailed review of these articles’ abstracts, based on the predefined inclusion and exclusion criteria, resulted in the exclusion of 37 articles. This left 56 articles for full-text screening. In the full-text review, six articles were excluded due to failing to meet one or more critical inclusion criteria. Specifically, these articles either did not provide sufficient empirical data on international students’ mental health during the COVID-19 pandemic or focused primarily on non-mental health outcomes. Ultimately, 50 articles were included in the final thematic analysis, as listed in Table 1.

### 2.5. Thematic Analysis Framework

To analyze the results of the 50 studies, a thematic analysis was conducted, adhering to the six-phase framework for psychological research outlined by Braun and Clarke [65]. This approach was chosen for its effectiveness in identifying, analyzing, and reporting patterns within various data types, ranging from qualitative to quantitative. This makes this approach particularly well suited to synthesizing and interpreting the multifaceted themes that emerge from the wide range of studies included in this review of mental-health-related literature. The specific steps involved in thematic analysis were as follows:

1. Familiarization with the data: The selected studies were examined closely to ensure a proper understanding of the content. Notes were taken on recurring concepts related to international students’ mental health during the pandemic.
2. Generating a coding scheme: A coding scheme was developed to identify notable aspects of the data related to the mental health impacts on international students during the pandemic. The codes captured key themes such as “social isolation”, “academic stress”, “financial insecurity”, and “discrimination”.
3. Identifying themes: The codes were then clustered into broader themes. Codes related to “financial stress” and “paying for groceries”, for instance, were clustered together to constitute a theme of “financial concerns and food insecurity”.
4. Reviewing themes: The themes were then reviewed and refined by the authors to ensure they accurately represented the data in a logically sound manner. At this stage, certain themes were merged or eliminated based on relevance. Cross-checking was performed between the themes and the original articles, based on the article summaries created, so that no key elements were overlooked.
5. Defining and naming themes: Upon the themes being firmly established, clear definitions were created for each theme to ensure fair characterization of the results being reviewed. For example, “social isolation” entailed feelings of loneliness, lack of peer support, and difficulty in maintaining social connections due to lockdowns and digital conversions. Each theme was then named succinctly for ease of reference in the final analysis and reporting.
6. Report production: Finally, the themes were interpreted and integrated into the narrative, providing a coherent synthesis of the literature. The analysis focused primarily on how these themes reflected the unique challenges faced by international students during the pandemic as they pertained to their mental health, offering insights and potential implications for student care.

In order to ensure the rigor and accuracy of the thematic analysis, the following strategies were employed. First, the authors engaged in triangulation of the thematic analysis, so as to ensure we reached an agreement in the coding and the rest of the analytical efforts. Second, the authors also engaged in ongoing conversations around emerging themes and challenge interpretations, ensuring balanced characterization and coverage. All in all, the coding and thematic processes proceeded smoothly, with very few, all very minor, discrepancies emerging.

Although this literature review primarily aimed to explore and synthesize existing research rather than to test specific hypotheses, we anticipated the following trends based on our preliminary understanding of the literature:

- Expectation 1: Specific factors, such as social isolation, financial insecurity, and discrimination, contributed to the relationship between the COVID-19 pandemic and mental health outcomes in international students.
- Expectation 2: International students experienced a greater decline in mental health during the COVID-19 pandemic compared to domestic students.
- Expectation 3: The negative mental health effects of the COVID-19 pandemic on international students persisted beyond the acute phase of the pandemic.

## 3. Results

### 3.1. Overview and Analytical Strategy

While the magnitude of the adverse mental health impact of the pandemic and the underlying factors varied somewhat across studies, all the papers reviewed in this study unequivocally highlighted the compromised mental health among international students. For instance, Tan and Sekercioglu [55] noted that 64.4% of international students studying in Canada reported experiencing moderate to severe anxiety and depression symptoms, while Alam et al. [1] revealed the following disturbing numbers based on a survey of 402 international students in China, reporting prevalent symptoms of depression (73.4%), anxiety (76.6%), stress (58.5%), insomnia (77.6%), psychological distress (71.4%), loneliness (62.4%), and fear (73.1%). In a study with international students in the US, Martirosyan et al. [49] found that 59% of respondents reported experiencing mental health challenges during the pandemic, with Reena et al. [52] reporting that 60% of participants from a comparable pool of students experienced heightened anxiety and depression. Finally, Um and associates [58] reported the disturbing figure that over 18% of the international students surveyed indicated having had self-harming thoughts, which is highly alarming. While the variability in the health assessment tools and research methods across studies necessitates a non-standardized interpretation of these figures, the preponderance of evidence unequivocally indicates a strong association between the COVID-19 pandemic and compromised psychological well-being among international students.

The conditions surrounding the compromised psychological well-being manifested primarily in such forms as depression, anxiety, and insomnia—and any combination thereof [1,40]. To meaningfully interpret and synthesize the 50 relevant articles reviewed in this study, we conducted a thematic analysis in a nuanced manner to capture specific features that are critical to the analysis. These modifications were primarily due to the locations of the universities (e.g., students studying in Turkey versus the United States) and the specific target student populations (e.g., all international students at American universities versus Chinese nationals attending American universities), as appropriate. This approach allowed for the identification and exploration of themes across different settings within the context of the pandemic, providing a more comprehensive understanding of the research findings.

Additionally, given that the themes often overlapped, it is crucial to recognize that these themes are closely intertwined and in no way independent of one another, necessitating a more contextualized discussion. For example, while the abrupt conversion to online learning and virtual academic advising was noted as a major stressor in the academic thematic domain, the same conversion was frequently cited as a key stressor under the theme of social isolation, as well [66]. Finally, while international students perennially face challenges such as language acquisition and cultural adaptation in their host countries [3,4], the literature reviewed here, naturally, focused on stressors arising from the pandemic exclusively. Therefore, these ongoing challenges were typically mentioned only in relation to the pandemic’s impact. For example, limited English proficiency was occasionally cited as a contributing factor to the confusion and frustration international students experienced navigating fluctuating US student visa policies during the pandemic [12,47]. Consequently, we opted not to treat the broader, pre-existing set of challenges faced by international students—such as adjusting to the new culture—as a distinct theme in this thematic analysis. By conducting the analysis in a contextualized manner, we could systematically explore how various factors relate to themes in different circumstances. This approach, in short, enhanced the overall rigor and depth of the qualitative analysis.

**Theme 1.** *Academic and professional challenges*

While not unique to international students, academic and professional challenges associated with the abrupt conversion to online instruction and student services during the pandemic emerged among the core themes in all the literature reviewed. Relatively early in the process, educational institutions around the world shifted to online instruction practically overnight to deter the spread of COVID-19 [31]. Specifically, across the 50 studies, the sudden shift to online learning was consistently reported as a major challenge for international students. Many students struggled with the technical aspects of online platforms, citing difficulties in accessing reliable internet, unfamiliarity with digital tools, and a lack of structured support from faculty [38,59,63]. For example, Tan and Sekercioglu [55] reported that 64.4% of international students in Canada experienced moderate to severe anxiety due to academic uncertainty. Similarly, Alam et al. [1] found that students in China reported high levels of academic stress, with 73.4% of respondents identifying academic challenges as a primary source of their mental distress.

In addition to academic concerns, many studies emphasized the professional impact of the pandemic. Internships, lab work, and field placements—critical components required for graduation and career progression—were often suspended or canceled, leaving students in limbo [1,41,43]. Kwak et al. [43] highlighted that international students in particular faced significant setbacks in securing post-graduation employment opportunities, especially as many destination countries imposed hiring freezes or reduced job availability. These professional disruptions compounded the uncertainty around academic progress, creating a cycle of anxiety for many students.

While some studies noted that domestic students also faced similar academic and professional challenges, the visa requirements specific to international students, which hinge heavily on maintaining steady academic progress, made the impact on this group disproportionately severe [51,55]. The importance of visa status was a recurring concern, with several studies [12,35,37,47,48,51] highlighting the additional layer of pressure faced by international students as they navigated both academic expectations and legal residency requirements during the pandemic, and this constitutes the second theme in the current analysis.

**Theme 2.** *Visa and residency concerns*

The COVID-19 pandemic brought about a wave of uncertainty for international students, particularly concerning their ability to maintain their visa or residency status. This was especially prominent in studies based in the United States [12,35,37,47,48,51], with some emphasis drawn in Australia [41], the UK [6], and Canada [55]. In the US, the July 2020 announcement requiring international students to attend in-person classes or face deportation—despite the reality that most universities did not offer in-person classes—triggered widespread confusion and distress [67]. Most US universities had already announced their intent to continue remote instruction for the fall semester, leaving international students in a precarious situation, unsure of their ability to remain in the country [47].

Lynch et al. [47] documented the frantic efforts of international students to navigate this unexpected challenge, ostensibly without viable solutions. Many students explored the daunting possibility of transferring to the few institutions offering in-person classes, despite the logistical and financial hurdles involved. The situation was further complicated by travel bans and restrictions that prevented some students from traveling and re-entering the country, even if they successfully registered for in-person classes [39]. Ke et al. [39] highlighted the plight of students who had left their host country before the widespread travel bans and subsequently faced significant barriers in their efforts to complete their studies.

Additionally, King and colleagues [41] uncovered an unfortunate coincidence for students from China studying overseas: the swift global spread of COVID-19 infections roughly coincided with the Lunar New Year celebration week in China in early 2020. As a result, some Chinese nationals had temporarily returned to China to celebrate with their families in the final week of January. Due to the rapid spread of the virus and corresponding travel restrictions—particularly those involving travelers from China, where the virus presumably originated [68,69]—many found themselves unable to return to Australia to resume their studies as planned. Although the shift to online instruction was systematically implemented within weeks, the academic uncertainties persisted, adversely impacting the psychological well-being of these students. Unsurprisingly, overall, concerns about remaining in good academic standing and graduating as planned have been cited as major contributors to depression, anxiety, and other negative effects among international students [41,44,56].

Travel restrictions persisted long after the shift to online learning began subsiding, disrupting academic continuity for many students stranded away from their universities. This uncertainty surrounding visa policies and travel restrictions fueled students’ anxiety and left many apprehensive about their future in their host countries.

**Theme 3.** *Social isolation and loss of support networks*

Social isolation emerged as another dominant theme across the literature, with students consistently reporting that the lack of in-person interactions took a significant toll on their mental health [13,15,23,59,70]. The transition to remote learning, coupled with government-imposed lockdowns, curtailed opportunities for social engagement, leaving many students feeling adrift from their peers, faculty, and support networks.

International students, as Collins [18] observed, typically rely on a diverse network of formal and informal support systems—academic advisors, peer groups, and cultural organizations—to navigate the complexities of life in their host country. The abrupt shift to remote interactions disrupted these vital connections, depriving students of crucial resources needed to cope with the challenges of studying abroad during a pandemic. The physical distance between students and their families further amplified feelings of isolation, as travel restrictions prevented many from returning home [34].

While the psychological impact of social isolation was particularly pronounced in China, where the stringent and closely monitored lockdown measures enforced persisted longer than in many other countries [23,64], this theme resonated across geographical boundaries. Relevant studies were conducted in Australia [18,19,20,39,41], Canada [44,55,64], Germany [34], Hungary [24], the Netherlands [42], Poland [25], Portugal [11], Taiwan [57], the United Kingdom [6,13,26], and the United States [6,12,26,35,37,43,44,47,48,50,51,53,54,58,59].

Two studies focusing upon international students in China provided compelling data, suggesting that the mental health consequences associated with social isolation, such as depression, anxiety, and insomnia, may be mitigated through physical exercise [32,45]. Interestingly, however, another study found that engagement in exercise was not predictive of psychological well-being among international students in Japan during the pandemic [33]. These contrasting results indicate that further research is necessary to ascertain the effectiveness of physical exercise in promoting mental health in similar contexts. Consequently, a more comprehensive examination of the available data is warranted to draw definitive conclusions.

**Theme 4.** *Living arrangements*

Living arrangements were another key factor influencing the mental health of international students during the pandemic. Studies showed mixed results regarding the impact of living with others versus living alone. Some studies reported that living with others provided students with essential social support, which helped mitigate feelings of isolation [15,22,39]. For example, Dong et al. [35] found that students living in shared housing reported lower levels of loneliness and higher levels of emotional support compared to those living alone.

However, other studies found the opposite, with students in shared living arrangements expressing concerns about health risks and difficulties in maintaining social distance [1,11,34,40]. These students reported increased anxiety about contracting COVID-19 from roommates or housemates, particularly in situations where shared spaces like kitchens and bathrooms were involved. In some cases, the tension of cohabiting during the pandemic led to conflicts and strained relationships, which further contributed to students’ mental distress.

As discussed in more detail in a later section, the variability in findings suggests that the impact of living arrangements on mental health may depend on individual circumstances, including the size and setup of shared spaces, the level of communication among housemates, and students’ pre-existing mental health conditions.

**Theme 5.** *Financial and food insecurity*

While financial hardship and food insecurity have long been recognized as significant stressors for university students, particularly in North America [71,72,73], the COVID-19 pandemic exacerbated these challenges, profoundly impacting the well-being of international students. Across the literature, a consistent theme emerged: international students faced considerable difficulties maintaining their financial stability and steady access to food during the pandemic [2,11,12,18,19,20,21,22,26,27,47,48,49,56,64]. Before the pandemic, many students relied on part-time jobs in sectors like hospitality and retail, which were severely impacted by lockdown measures [74]. Consequently, these students found themselves without a steady income, facing financial strain and uncertainty.

Food insecurity emerged as a particularly critical issue, especially in contexts where lockdowns disrupted supply chains and limited access to affordable food sources and public transportation. Mihrshahi et al. [19] observed that the combination of financial strain and logistical barriers to food access created a dual burden for international students who did not have family and relatives to rely on in their immediate neighborhoods, exacerbating their mental health challenges. Another study similarly linked food insecurity to heightened stress and anxiety, with students expressing concerns about meeting their basic needs during the pandemic [49]. Beyond the immediate financial strain, the uncertainty about the future—whether students would be able to return to part-time work or receive financial assistance—contributed significantly to their psychological distress. The lack of a clear timeline for recovery in many industries further fueled feelings of instability and fear [12].

**Theme 6.** *Health concerns for self and loved ones*

The COVID-19 pandemic cast a long shadow of health-related anxiety over international students. Many expressed profound fear not only for their own well-being [1,15,18,21,26,27,35,36,42,46,47,53,60,63] but also for the health of their families in their home countries [26,32,46,47,52,54]. This fear was palpable in the data, with Martirosyan et al. [49] reporting that 59% of international students in the US expressed anxiety about contracting the virus. Similarly, Reena et al. [52] found that 60% of students voiced heightened concern for the health and safety of family members back home.

Beyond the immediate threat to physical health, a pervasive fear of social stigma emerged from the shadows. Several studies revealed that students dreaded the social repercussions of a COVID-19 diagnosis. This fear was particularly acute among East Asian students, who were already navigating the treacherous terrain of discrimination due to the virus’s origins in China [58]. The students surveyed described how the stigma surrounding COVID-19 infection led to feelings of shame, isolation, and a profound sense of being “othered”, further compounding their psychological distress.

**Theme 7.** *Actual, anticipated, or vicarious discrimination and xenophobia*

The COVID-19 pandemic brought to the forefront the harsh reality of discrimination faced by international students, particularly those of East Asian descent. Across the literature, a recurring theme was the experience of actual, vicarious, and anticipated encounters with racial discrimination, compromising their psychological well-being [48,58,61,62]. Reena et al. [52] revealed the stark prevalence of this issue, reporting that 60% of East Asian students in their study had encountered some form of racial discrimination during the pandemic.

The psychological toll of such discrimination was profound. Many students described experiencing heightened levels of anxiety, depression, and a retreat into social withdrawal as a consequence of these encounters. In some instances, the mere anticipation of discrimination, even in its absence, cast a pall of fear and vigilance over international students’ lives [58,61,62,75]. The enduring nature of these psychological wounds remains a significant concern, as studies suggest that the scars of pandemic-related racism may linger long after the immediate crisis has subsided.

### 3.2. Summary of the Results

These seven themes, derived from 50 papers examining the experiences of international students during the COVID-19 pandemic, provide a comprehensive overview of the vulnerabilities experienced by this population. As anticipated, factors such as social isolation, financial insecurity, and discrimination were prominent contributors to the mental health challenges faced by international students, aligning precisely with Expectation 1. The support for Expectation 2, which posited that the adverse impacts of the pandemic would be more prevalent among international students compared to their domestic counterparts, was mixed and is described in greater detail in the following section. The longer-term consequences, articulated in Expectation 3, were not addressed in the reviewed literature, potentially due to the limited time that had elapsed since the pandemic’s end. The following sections provide a more in-depth discussion of the identified themes.

## 4. Discussion

The results of this literature review have thus far highlighted the multifaceted challenges faced by international students during the COVID-19 pandemic. Seven core themes emerged: academic and professional challenges; visa and residency concerns; social isolation and loss of support networks; living arrangements; financial and food insecurity; health concerns for self and loved ones; and actual, anticipated, or vicarious experiences of discrimination. This supports our Expectation 1, specific factors, such as social isolation, financial insecurity, and discrimination, contributed to the mental health outcomes in international students. These themes reveal that the pandemic likely exacerbated pre-existing vulnerabilities, such as navigating daily life in a foreign land and, often, language barriers, while also introducing new and unfamiliar stressors like travel bans and restrictions impacting student visa eligibility. In this section, we will contextualize these results within the broader context of international student experiences, drawing attention to both the unique pressures they faced and the opportunities for institutional support moving forward.

Based on the challenges and vulnerabilities identified through our thematic analysis, we propose a series of targeted interventions designed to mitigate the adverse psychological impact of the COVID-19 pandemic on international students. These suggestions are grounded in the lived experiences of international students as reflected in the reviewed literature and aim to provide practical and actionable strategies for institutions to support this vulnerable population.

**Theme 1.** *Academic and professional challenges*

The studies unanimously noted that the transition to online learning was a critical stressor for international students in a multitude of ways. This disruption not only impacted students’ academic progress but also caused concerns about jeopardizing their student visa status, which exclusively hinges on maintaining a full course load and meeting academic performance standards. Studies on online learning more generally have demonstrated that student engagement is significantly reduced in remote environments, particularly for students who rely on physical interactions with faculty and peers [38,59]. The challenges faced by international students in this regard align with previous research, which has shown that the shift to online learning disproportionately affects students from marginalized or non-native backgrounds [56].

International students faced unique setbacks as the pandemic disrupted essential components of their academic and professional development. Laboratory work, internships and practica, practical experience opportunities, and job prospects—often integral parts of on-time degree completion and maintaining their student visa status—were abruptly put on hold or canceled altogether. This, according to the literature reviewed, sparked significant anxiety not only about their student visa maintenance but also about future career trajectories, as these students faced uncertainty about fulfilling academic requirements and remaining within the parameters of their student visas, which decidedly require full-time enrollment [43]. These findings are consistent with studies on international student experiences during economic downturns, which show that uncertainty around future opportunities is a significant contributor to mental health challenges [1,43].

To mitigate the psychological damage experienced by international students due to academic and professional challenges as outlined under this theme, institutions of higher education may consider the following targeted interventions in response to the concerns cited under this theme:

1. Enhanced communication: Establish robust, transparent, regular, and timely communication channels to keep international students informed about changes in student visa requirements, academic policies, graduation requirements, and available support services. Regular updates and clear guidelines can help reduce uncertainty, frustration, and anxiety.
2. Flexible academic policies: Introduce and widely disseminate more flexible academic policies that accommodate the academic challenges that international students may face, such as extended deadlines and pass/fail options, keeping in mind that international students’ eligibility to stay in their destination countries usually hinges heavily on their successful and uninterrupted academic progress as full-time students. These policies, when announced swiftly, can help students manage stress linked to their academic responsibilities more effectively during uncertain times without diluting their learning experiences.
3. Career services, professional development, and online learning support: Enhance career services to support students in finding internships and job opportunities, even remotely. This includes virtual job fairs, resume-building workshops, and networking events. Since many international students find it essential to secure internships or employment upon graduation in the host country, this is critical in addressing the concerns widely cited by these students. Additionally, offer comprehensive training and technical support for online learning platforms to help students navigate the environment effectively, alleviating the frustration associated with remote learning. Both faculty and administrative staff should be briefed on these matters, so as to provide cohesive and comprehensive student support for international students.

By implementing these strategies, institutions can help alleviate some of the psychological burdens faced by international students and promote a more supportive and resilient academic environment during and beyond the pandemic.

**Theme 2.** *Visa and residency concerns*

While overlapping with the first theme identified earlier concerning academic and professional challenges, the uncertainty surrounding visa status constituted a major source of stress for international students during the pandemic, particularly in countries like the United States where visa policies continued to evolve [12,35,37,47,48,51]. In 2020, most US universities had no firm plans for transitioning back to in-person classes and public opinion was still very much against the reopening of schools [76,77]; in addition, the few that provided in-person classes were unable to accommodate all students due to space limitations and instructor availability. As a result, this proposal elicited significant and widespread concerns, confusion, and chaos [12].

Perhaps in the face of such backlash, the US Immigration and Customs Enforcement soon supplemented the policy with a recommendation that international students seek transfers to universities offering in-person classes [56]. This recommendation was made presumably in an attempt to mitigate the issues raised and address the needs of international students who were disproportionately affected by the limited availability of in-person instruction [47]. Nevertheless, one could argue that this policy and the iterations that followed (e.g., the recommendation that international students transfer to the few universities that offer in-person instruction, with only a few weeks before classes were set to begin) were entirely impractical, as the majority of American universities were set to begin their fall session by the following month and the deadlines for transfer applications had long passed. In other words, this timing rendered the proposed visa policy and subsequent updates unfeasible, as international students had insufficient time to make alternative arrangements. It may thus be feasible to allege that international students faced these challenges without any viable solutions. Although policies were reportedly less restrictive, those studying in other countries, such as Australia [41], the UK [6], and Canada [55], too, reported similar dilemmas and struggles around student visa statuses. This precarious situation was bound to have a profound impact on their mental health, as students feared deportation or, at a minimum, being forced to interrupt their studies. These results align with previous research showing that legal uncertainties are a key source of anxiety for international students.

At the same time, international students reportedly felt frustrated with their own institutions for failing to respond swiftly to policy-related matters directly impacting their academic standing and continuation as students; consequently, it is unsurprising that the lack of transparency and action by universities has been cited as a key factor contributing to the decline in international student mental health, in addition to the opaque government policies [12,35,47,48,50,51].

To alleviate the psychological burdens experienced by international students due to practical concerns related to this theme, visa and residency status, institutions of higher education should consider implementing the following targeted interventions, although there is an overlap between the suggestions made for these and Theme 1 discussed earlier:

1. Develop clear and proactive communication strategies specifically aligned with evolving government policies and the often chaotic flow of information regarding such relevant issues as visa regulations and travel restrictions, along with other information of particular interest to international students—all consolidated in one location (such as a special website). Ensuring that international students receive timely and accurate information in response to these changing circumstances can alleviate anxiety and uncertainty related to their legal status and academic progress—in clear language.
2. Advocating for realistic, flexible and equitable student visa policies: Universities should actively engage with government bodies to advocate for more realistic, flexible and equitable student visa policies that recognize the unique challenges faced by international students during global crises. This advocacy should explicitly oppose measures such as those proposed in 2020 in the US, which mandated in-person course enrollment for international students to maintain their visa status, regardless of public health concerns or institutional capacity. Such policies disregard the realistic needs and circumstances of international students, potentially jeopardizing their academic progress. Universities can support students by providing documentation and evidence of academic progress and individual needs to immigration authorities, facilitating a more adaptable and compassionate approach to visa and residency requirements. This advocacy requires long-term planning and commitment to ensuring that student visa policies are responsive to the realities of international education and prioritize the well-being and academic success of international students.
3. Specialized support services: Establish specialized support services focused on the legal and logistical challenges faced by international students. Providing resources such as legal advice on visa issues, assistance with travel arrangements, and guidance on navigating bureaucratic processes can help students maintain their residency status and academic standing more effectively.

These initiatives can target the unique challenges faced by international students, especially in host nations with stricter immigration policies, easing the psychological strain related to upholding their legal status and academic progress throughout and following these unusual circumstances.

**Theme 3.** *Social isolation and loss of support networks*

Social isolation was one of the most pervasive issues reported by international students during the pandemic, and it is well established in the literature that social support is critical to students’ well-being [78,79]. The transition to remote learning severed many of the formal and informal networks that international students rely on, leaving them disconnected from academic advisors, peer groups, and cultural organizations [18]. This loss of connection was particularly damaging to mental health, as students reported feelings of loneliness and alienation, as discussed earlier in the Results section.

These results are consistent with research on social isolation among international students, which has long identified the importance of building strong support networks in the host country [13,15,27,45,53]. The pandemic amplified these challenges, as lockdown measures prevented students from accessing in-person resources and participating in cultural or social events that could have mitigated feelings of isolation [34].

Two studies focusing on international students in China provide compelling data, suggesting that the mental health consequences associated with social isolation, such as depression, anxiety, and insomnia, might have been mitigated through physical exercise [32,45]. Interestingly, however, another study found that engagement in exercise was not predictive of psychological well-being among international students in Japan during the pandemic [33]. One could speculate that the exceedingly tightly implemented lockdowns in China [23,46] versus the relatively relaxed practice in other countries, including Japan, may explain the discrepancy here; however, these contrasting results indicate that further research is necessary to ascertain the effectiveness of physical exercise in promoting mental health in similar contexts. Consequently, a more comprehensive examination of the role of physical activities vis-à-vis social isolation and mental health is, thus, in order.

To address the psychological impacts of social isolation, activity restrictions, and lack of human connection and support, institutions of higher education should consider implementing the following targeted interventions:

1. Fostering virtual communities and affinity-based support networks when face-to-face interactions are difficult: Develop robust virtual platforms that facilitate social interactions and peer support among international students, even when in-person events are impractical. This intervention is particularly promising, as recent research has demonstrated how digital platforms could serve to promote mental health among university students [16]. These platforms should support the formation of affinity-based groups based on shared hobbies, professional interests, support needs, race/ethnicity, culture, and other relevant dimensions. Informal and formal virtual events, online clubs, and peer mentoring programs can help mitigate feelings of isolation by fostering a sense of community and belonging tailored to the diverse backgrounds and interests of students. Although the findings around engagement in physical exercise are mixed in the current context, basing social interactions around exercising could serve to minimize the adverse psychological consequences of social isolation.
2. Improving mental health care access and reducing stigma: Establish comprehensive mental health services that are easily accessible and culturally responsive, not only in its practice but also in reach-out efforts. Provide virtual counseling, mental health hotlines, with multilingual considerations, and peer support groups to ensure that students receive the help they need. Additionally, implement campaigns to reduce the stigma associated with seeking mental health care, promoting a supportive environment where students feel comfortable reaching out for help.

These approaches can serve to lessen the mental health effects of social isolation and limited activities, creating a more nurturing and resilient atmosphere for international students in times of social deprivation.

**Theme 4.** *Living arrangements*

The impact of living arrangements on students’ mental health during the pandemic varied considerably, with some students reportedly having benefitted from shared living [15,22,39] situations while others experienced increased stress due to concerns about health risks and privacy [1,11,34,40]. Previous studies have shown that living arrangements can either mitigate or exacerbate mental health issues, depending on the level of social support and interpersonal conflict within shared spaces, pointing to the need to consider individual preferences and circumstances [15].

The results of this review suggest that the impact of living arrangements on mental health is closely tied to the dynamics within the living contexts, although the results seem mixed. All things considered, the following recommendations could be made to alleviate issues concerning living conditions.

1. Universities should provide flexible housing options for international students, including single-occupancy rooms for those who are concerned about health risks and prefer to live alone. Similarly, group living options with enhanced health and safety protocols should also be explored to accommodate students who prefer to avoid living alone.
2. Institutions should also consider offering virtual social activities and mental health resources, especially to students living alone, to reduce the negative impact of isolation. Tailored housing support based on individual needs: Given the mixed research findings regarding the benefits of living alone versus living with others, provide flexible housing options that accommodate the potentially varied preferences of international students. Offer both shared and private living arrangements and ensure that students are sufficiently well informed about these options to make choices that best suit their individual needs and expectations. Commuter universities, too, could establish housing support programs to address international students’ off-campus living needs, preferences, and expectations.
3. Provide the means for safe and healthy living environments: Inform and implement measures to promote safe and healthy living conditions in shared accommodation, such as clear guidelines for regular sanitation, suggested methods of preventing the spread of infections, and providing personal protective equipment (PPE; such as disposable gloves, disinfectant cleaners, room dividers, and face shields). These measures can help alleviate concerns about health risks associated with shared living spaces.

By implementing these strategies, institutions can better address the varied impacts of living arrangements on the mental health of international students, thus promoting a more supportive and adaptable housing environment during and beyond the pandemic.

**Theme 5.** *Financial and food insecurity*

Financial concerns were a major source of stress for international students during the pandemic, with many students losing part-time jobs and struggling to meet basic living expenses [2,11,12,18,19,21,22,26,27,39,47,48,49,56,64]. Food insecurity, too, emerged as a significant issue, as lockdowns and business closures limited access to affordable food and public transportation [19]. These results align with existing research on the financial vulnerabilities of these students, who are often excluded from government financial aid programs and rely heavily on precarious forms of employment [74,80].

The psychological toll of financial insecurity cannot be overstated, as students reported high levels of anxiety and stress related to their ability to meet basic needs. It is feasible to argue that the combination of financial strain and limited access to food was particularly detrimental to students’ mental health, as it compounded the effects of social isolation and academic stress. To address the mental health impacts related to financial concerns and food insecurity, institutions of higher education should consider the following interventions:

1. Actively disseminating information on financial assistance, food-access programs, and flexible tuition and fee payment options: Provide clear and comprehensive information on potential assistance available to international students, including scholarships, grants, external funding sources, and community programs. Additionally, offer flexible payment deadlines, fee waivers, and installment plans to help students manage their financial obligations more effectively during crises.
2. Ensuring access to food and basic necessities: Establish food pantries and meal programs on campus that are accessible to students, even during lockdowns, to the greatest extent possible. Partner with local food vendors and transportation services to ensure students have reliable access to affordable and nutritious food, regardless of their financial situation or logistical challenges.
3. Financial literacy workshops: Offer workshops tailored to the specific financial challenges and needs of international students. These workshops can cover topics like budgeting, negotiating with landlords, managing debt, understanding the US credit system, accessing banking services, and navigating financial aid options.
4. Connect with cultural organizations: Partner with cultural organizations and consulates that serve international student populations. These organizations might offer additional financial or food-access resources or support programs specifically for students from certain countries.

These measures can address the financial hardships and food insecurity that international students face, fostering a stronger and more supportive campus environment both now and in the future.

**Theme 6.** *Health concerns for self and loved ones*

Health concerns were a significant contributor to the psychological distress experienced by international students during the pandemic. Many students expressed fear of contracting COVID-19 [1,15,18,21,26,27,35,36,42,46,47,53,61,63], as well as anxiety about the health and safety of their family members back home [26,32,46,47,52,54].

East Asian students, in particular, reported significant fear of stigmatization and discrimination related to the origins of the virus in China, further compounding their health-related anxiety; for instance, fear that contracting the virus would serve to confirm the stereotype [81]. These findings align with research on the intersection of race, health, and mental well-being, which has identified racialized health stigma as a significant stressor for minority groups during public health crises [82,83].

Addressing the health concerns of international students, both for themselves and their loved ones, requires targeted interventions by institutions of higher education. Based on the literature reviewed here, the following strategies could help mitigate these psychological impacts.

1. Providing comprehensive health information and preventive strategies: Ensure that international students have access to accurate and practical information about COVID-19 and other health concerns, especially recognizing that they may be overwhelmed with the amount of information gathered through a wide range of sources. This should include succinct guidance on preventive measures, realistic expectations about symptoms, and updates on vaccination and testing. Additionally, offer online health workshops and telehealth services to provide ongoing support and information, especially given that many international students are reluctant to seek such help.
2. Reducing stigma and promoting mental health support: Implement campaigns to reduce the stigma associated with contracting COVID-19 and promote a supportive environment. This includes reach-out efforts by mental health support services, such as counseling and peer support groups, that are sensitive to the cultural and social needs of international students.
3. Facilitating communication with family and support networks: Inform international students about effective strategies for maintaining regular communication with their families and support networks to minimize the psychological toll. Provide resources, such as information on free or subsidized internet access and communication tools, and emphasize the importance of staying connected to reduce anxiety and stress related to the health of their loved ones.
4. Health insurance navigation: Assist international students in understanding their health insurance plans, navigating the healthcare system, and finding affordable healthcare options off-campus if needed. This could involve workshops, individual consultations, or online resources.

Through implementing these strategies, institutions could help address the health-related concerns of international students, promoting a more supportive and resilient environment during and beyond the pandemic.

**Theme 7.** *Actual, anticipated, or vicarious discrimination and xenophobia*

Experiences of discrimination and xenophobia were pervasive among international students during the pandemic, particularly those of East Asian descent. Students reported both overt and subtle forms of discrimination, which significantly impacted their mental health. These results are consistent with studies on the racialization of pandemics, which have shown that groups of color are often scapegoated during public health crises [75,84,85]. The psychological effects of discrimination appear to have been profound, with students reporting increased anxiety, depression, and social withdrawal as a result of their experiences, as discussed earlier. The long-term impact of such discrimination may potentially be particularly disconcerting, as studies suggest that the mental health consequences of racialized stigma may persist long after the pandemic has subsided [61,62].

To effectively address the psychological impacts of actual, vicarious, or anticipated discrimination among international students from specific cultural groups—in this case, East Asian and East Asian-appearing students—institutions of higher education should consider the following strategies.

1. Promoting cultural competency and raising awareness about xenophobia: Develop and disseminate information pertaining to comprehensive cultural competency designed to inform the university community about the harmful effects of racism and xenophobia, as well as the misdirected nature of hate toward specific groups. These programs should promote inclusivity, provide tools for recognizing and combating subtle forms of bias, and raise awareness about the specific challenges, in the case of the COVID-19 pandemic, faced by East Asian students during public health crises.
2. Establishing support networks and facilitating open conversations: Universities should create a multifaceted support system. This begins with establishing clear and accessible reporting channels for incidents of discrimination and xenophobia, ensuring that students feel safe and empowered to report these incidents without fear of retaliation. Once reported, incidents must be followed up with robust support networks specifically for students from impacted backgrounds, such as those from East Asian communities. These networks can offer peer support, counseling services, and safe spaces for students to discuss their experiences and process their emotions. While local governments and community centers may offer similar resources, it is crucial that universities integrate these supports into their own systems to ensure accessibility, relevance, and a sense of institutional accountability. Beyond reactive support, universities should proactively facilitate open conversations and workshops on effectively dealing with xenophobia and hostility. These programs should empower students with strategies to navigate and cope with these challenges, fostering resilience and self-advocacy.

Adopting these strategies can alleviate the psychological effects of discrimination and cultivate a more inclusive and supportive atmosphere for international students, both during the pandemic and in the future.

### 4.1. Additional Nuances and Considerations

Although some matters of interest appeared only sporadically and were thus excluded from our thematic analysis, we believe the following may deserve further exploration. For instance, some studies reviewed here yielded mixed results regarding the psychological distress experienced by international students compared to their domestic counterparts. While several studies reported that international students experienced greater psychological distress vis-à-vis the pandemic in a number of countries across continents compared to their domestic peers [13,18,19,20,24,42], a study in the nation of Georgia reported that they were psychologically healthier than their domestic counterparts [21]. Thus, mixed results were obtained in relation to our Expectation 2, international students experienced a greater decline in mental health during the COVID-19 pandemic compared to domestic students. This inconsistency highlights the complexity of the issue and suggests that the psychological impact of the pandemic may perhaps be influenced by a variety of contextual and confounding factors, such as the nature of the student bodies as well as the host cultures. As such, further explorations would allow us to gain a more thorough understanding of the dynamics revealed here.

In addition to the variations between international and domestic students, sex emerged as another interesting point of potential inquiry. While the majority of studies that alluded to sex-related results indicated a more profound impact of the pandemic on the mental health of female international students compared to their male counterparts across a variety of nations [15,21,24,26,32,52,55], other studies reported diametrically opposed results among international students in China and South Korea [1,40]. These contrasting results highlight the necessity for further in-depth research to unravel the intricate interplay among sex, gender roles, and the mental health experiences of international students during global crises. Such investigations should also consider the potential influence of both students’ countries of origin and the cultural context of their host countries.

Finally, while increased familiarity with the language and culture of the host nations was generally linked to more positive mental health outcomes, there was a notable exception. Specifically, cultural adaptation in the host countries was generally related to healthier psychological conditions [25,41,64]. This observation aligns with previous research suggesting that discomfort within the host culture can lead to additional stressors, frequently noted as a key challenge faced by international students. Curiously, however, one study on international students studying in South Korea [40] revealed that increased fluency in the Korean language was associated with less favorable mental health outcomes. Kim and Kim argue that, perhaps, having access to the local Korean media, in addition to the media outlets in their native or other preferred languages, overwhelmed these strong multilingual speakers.

The interplay among varying factors, such as the “local culture” of the host countries and potentially unique features of the student body, has yet to be examined in the current context. The link between cultural adaptation in the host nations and mental health in times of crisis, therefore, needs to be further investigated, particularly to inform effective support strategies for international students during challenging periods. These contrasting results underscore the importance of considering a range of variables and contexts when examining the psychological well-being of students during crises. These results emphasize the need for nuanced and contextualized interventions that address the diverse needs of both international and domestic students during times of crisis.

### 4.2. Limitations and Future Directions

This review underscores the significant mental health challenges faced by international students during the COVID-19 pandemic. While the results suggest the detrimental impact that the pandemic had on their well-being, it is crucial to acknowledge the limitations of the existing research. First, the dearth of pre-post comparative studies limits the ability to definitively determine whether the observed mental health difficulties were solely attributable to the pandemic or if pre-existing vulnerabilities were exacerbated. While one study [15] provided some evidence of increased mental health challenges during the pandemic, the lack of broader comparative data restricts the generalizability of its findings. Furthermore, the reliance on self-reported data may have introduced bias, particularly when discussing a highly unusual global health crisis, potentially influencing the reported distress levels. The heterogeneity of the methodologies employed across studies further complicates direct comparisons and the development of definitive conclusions regarding the universal impact of the pandemic on this population. Future research employing longitudinal designs could address these limitations, providing insights into both pre- and post-pandemic mental health trajectories.

Critically, the absence of studies that directly track mental health outcomes over time, including those with distal follow-up assessments, limits our understanding of the long-term mental health consequences for international students. While the studies included in this review captured self-reported experiences and challenges during or shortly after the pandemic, and some alluded to the potential for lasting adverse mental health impacts [15,61], none directly examined the enduring effects or the possibility of prolonged psychological distress. Consequently, our Expectation 3—that the negative mental health effects of the COVID-19 pandemic on international students persisted beyond the acute phase—remains unaddressed in this review. This gap is perhaps expected, however, as the pandemic only occurred recently, and the implementation of longitudinal designs would require more time to yield robust findings. Moreover, the pandemic coincided with other significant stressors, including heightened political tensions and xenophobic rhetoric, particularly targeted towards individuals of Asian descent. Disentangling the specific impact of the pandemic from these confounding factors is challenging, raising questions about the extent to which the observed mental health difficulties were uniquely caused by the pandemic or influenced by a complex interplay of socio-political factors.

Finally, future research should systematically evaluate the effectiveness of intervention efforts that institutions of higher education may have implemented during the COVID-19 pandemic to support the mental health and well-being of their international students. These interventions may include, but are not limited to, those proposed in this review. Such investigations should examine their impact on various mental health outcomes and identify factors that may influence their success. Such research will provide valuable evidence to guide institutions in developing and implementing targeted support programs that promote the well-being and academic success of international students, fostering a more inclusive and resilient global community. Addressing these limitations is essential for accurately characterizing the mental health needs of international students and developing effective, targeted support strategies.

## 5. Conclusions

The results of the current literature review unequivocally highlight the significant mental health challenges faced by international students during the COVID-19 pandemic. The thematic analysis revealed seven key areas of concern: academic and professional challenges, visa and residency concerns, social isolation, living arrangements, financial and food insecurity, health impacts for self and loved ones, and actual, anticipated, or vicarious discrimination and xenophobia. Understanding these themes in depth is crucial to providing effective care and support for international students, especially given the specific needs each student may have depending on their backgrounds and preferences. The nuances associated with each theme emphasize the need for tailored interventions that address the unique challenges faced by this population, as suggested under each theme. Institutions of higher education must recognize the interconnectedness of these issues and adopt comprehensive strategies to mitigate their impact.

Taking good care of international students is not just a moral obligation but a strategic investment in a future where individuals from all walks of life can coexist peacefully, productively, and sustainably [28,29,86]. These students enrich our campuses with wider ranges of knowledge, skills and perspectives, and they contribute to the academic, cultural, and economic vitality of our communities. By addressing their unique needs and ensuring their well-being, we can foster a climate of intercultural collaboration that benefits not only the international students themselves but also their domestic peers, faculty, staff, and the broader local community. In today’s global economy, further research into understanding and supporting international students is a crucial step toward building a more interconnected and resilient world.

## Figures and Tables

**Table 1:** Alphabetical list of the articles included in the current analysis.

Authors	Year	University Locations
Alam, M.D., Lu, J., Ni, L., Hu, S., and Xu, Y. [1]	2021	China
Al-Oraibi, A., Fothergill, L., Yildirim, M., Knight, H., Carlisle, S., O’Connor, M., Briggs, L., Morling, J. R., Corner, J., Ball, J. K., Denning, C., Vedhara, K., and Blake, H. [13]	2022	UK
Antwi, C. O., Belle, M. A., Ntim, S. Y., Wu, Y., Affum-Osei, E., Aboagye, M. O., and Ren, J. [32]	2022	China
Cairns, D., França, T., Calvo, D. M., and De Azevedo, L. F. [11]	2022	Portugal
Chen, Y., Shimamoto, K., Bando, T., and Tsuchiya, H. [33]	2024	Japan
Collins, C., Haase, D., Heiland, S., and Kabisch, N. [34]	2022	Germany
Collins, F. E. [18]	2021	Australia
Dong, F., Hwang, Y., and Hodgson, N. A. [35]	2023	US
Elemo, A. S., Ahmed, A. H., Kara, E., and Zerkeshi, M. K. [36]	2022	Turkey
Gao, N., Eissenstat, S. J., Wacha-Montes, A., and Wang, Y. [37]	2022	US
Ge, L. [38]	2021	Canada
Iftikhar, S., Perceval, G., Fu, Y., Zhou, C., and Cao, Y. [2]	2022	China
Jamshaid, S., Bahadar, N., Jamshed, K., Rashid, M., Imran Afzal, M., Tian, L., Umar, M., Feng, X., Khan, I., and Zong, M. [15]	2023	China
Jamshaid, S., Olorundare, A., Wang, L., Lo-Ngoen, N., Afzal, M. I., and Bibi, M. [27]	2022	China
Ke, T., Li, W., Sanci, L., Reavley, N., Williams, I., and Russell, M. A. [39]	2023	Australia
Kim, H. R., and Kim, E. J. [40]	2021	South Korea
King, J. A., Cabarkapa, S., Leow, F. H., and Ng, C. H. [41]	2020	Australia
Kivelä, L., Mouthaan, J., Van Der Does, W., and Antypa, N. [42]	2022	The Netherlands
Kwak, M., Kwon, E., Lo, L., and Jung, G. [43]	2024	Canada
Lai, A. Y., Lee, L., Wang, M., Feng, Y., Lai, T. T., Ho, L., Lam, V. S., Ip, M. S., and Lam, T. [26]	2020	UK and US
Lai, A. Y., Sit, S. M., Lam, S. K., Choi, A. C., Yiu, D. Y., Lai, T. T., Ip, M. S., and Lam, T. [6]	2021	UK and US
Lin, C., Tong, Y., Bai, Y., Zhao, Z., Quan, W., Liu, Z., Wang, J., Song, Y., Tian, J., and Dong, W. [44]	2022	US
Lu, G., Hu, S., Zhang, Y., Chen, J., Yuan, Y., Gong, X., and Zhang, Y. [45]	2022	China
Lu, L., Wang, X., Wang, X., Guo, X., and Pan, B. [46]	2022	China
Lynch, J., Gesing, P., and Cruz, N. [47]	2023	US
Maleku, A., Kim, Y. K., Kirsch, J., Um, M. Y., Haran, H., Yu, M., and Moon, S. S. [48]	2022	US
Martirosyan, N. M., Van De Walker, D., and Saxon, D. P. [49]	2022	US
Mbous, Y. P. V., Mohamed, R., and Rudisill, T. M. [50]	2022	US
Mihrshahi, S., Dharmayani, P. N. A., Amin, J., Bhatti, A., Chau, J. Y., Ronto, R., Turnip, D., and Taylor, M. [19]	2022	Australia
Nadareishvili, I., Syunyakov, T., Smirnova, D., Sinauridze, A., Tskitishvili, A., Tskitishvili, A., Zhulina, A., Patsali, M. E., Manafis, A., Fountoulakis, N. K., and Fountoulakis, K. N. [21]	2022	Georgia (the nation of)
Park, C., and Shimada, S. [12]	2022	US
Prasath, P. R., Xiong, Y., Zhang, Q., and Jeon, L. [51]	2022	US
Reena, I., Hebert, E., Das, K., Doe, R., Hebert, S., and Gope, N. [52]	2023	US
Reid, C., Beckstead, J., and Salinas-Miranda, A. [53]	2022	US
Rekenyi, V., Garbóczy, S., Szemán-Nagy, A., Al-Tammemi, A. B., Sayed-Ahmad, M., and Kolozsvári, L. R. [24]	2023	Hungary
Russell, M. A., Reavley, N., Williams, I., Li, W., Tarzia, L., Chondros, P., and Sanci, L. [20]	2023	Australia
Shek, D. T. L., Dou, D., and Zhu, X. [22]	2022	Hong Kong
Song, B., Zhao, Y., and Zhu, J. [54]	2020	US
Tan, S., and Sekercioglu, F. [55]	2022	Canada
Tan, Y., Wu, Z., Qu, X., Liu, Y., Peng, L., Ge, Y., Li, S., Du, J., Tang, Q., Wang, J., Peng, X., Liao, J., Song, M., and Kang, J. [56]	2022	EU, Hong Kong, Macao, North America, Oceania, and Taiwan
Tran, T. X., Vo, T. T. T., and Ho, C. [57]	2023	Taiwan
Um, M. Y., Maleku, A., Haran, H., Kim, Y. K., Yu, M., and Moon, S. S. [58]	2022	US
Wang, L. [59]	2023	Australia, Canada, EU, Hong Kong, Japan, Malaysia, UK, and US
Wilczewski, M., Gorbaniuk, O., and Giuri, P. [25]	2021	Poland
Xiong, Y., Prasath, P. R., Zhang, Q., and Jeon, L. [60]	2022a	US
Xiong, Y., Prasath, P. R., Zhang, Q., and Jeon, L. [61]	2022b	US
Yang, L., Kandasamy, K., and Na, R. [62]	2021	Canada
Younis, I., Longsheng, C., Zulfiqar, M. I., Imran, M., Shah, S. A. A., Hussain, M., and Solangi, Y. A. [63]	2021	China
Yu, L., Cao, Y., Wang, Y., Liu, T., MacDonald, A., Bian, F., Li, X., Wang, X., Zhang, Z., Wang, P. P., and Yang, L. [64]	2023	Canada
Yuan, L., Lu, L., Wang, X., Qu, M., Gao, Y., and Pan, B. [23]	2023	China

## Data Availability

The data supporting the findings of this review are available within the article. Table 1 provides an exhaustive list of the publications included in this analysis.
